# Development of a multivariable prediction model for identification of patients at risk for medication transfer errors at ICU discharge

**DOI:** 10.1371/journal.pone.0215459

**Published:** 2019-04-30

**Authors:** Liesbeth B. E. Bosma, Nienke van Rein, Nicole G. M. Hunfeld, Ewout W. Steyerberg, Piet H. G. J. Melief, Patricia M. L. A. van den Bemt

**Affiliations:** 1 Haga Teaching Hospital, Department of Clinical Pharmacy, Els Borst-Eilersplein CH, The Hague, The Netherlands; 2 Erasmus University Medical Center, Department of Hospital Pharmacy, CA, Rotterdam, The Netherlands; 3 Leiden University Medical Center, Department of Clinical Pharmacy and Toxicology, Leiden, The Netherlands; 4 Erasmus University Medical Center, Department of Intensive Care, CA, Rotterdam, The Netherlands; 5 Clinical Biostatistics and Medical Decision Making at Erasmus MC, Rotterdam and Leiden University Medical Center, ZA Leiden, The Netherlands; 6 Haga Teaching Hospital, Department of Intensive Care, CH, The Hague, The Netherlands; University of Notre Dame Australia, AUSTRALIA

## Abstract

**Introduction:**

Discharge from the intensive care unit (ICU) is a high-risk process, leading to numerous potentially harmful medication transfer errors (PH-MTE). PH-MTE could be prevented by medication reconciliation by ICU pharmacists, but resources are scarce, which renders the need for predicting which patients are at risk for PH-MTE. The aim of this study was to develop a prognostic multivariable model in patients discharged from the ICU to predict who is at increased risk for PH-MTE after ICU discharge, using predictors of PH-MTE that are readily available at the time of ICU discharge.

**Material and methods:**

Data for this study were derived from the Transfer ICU Medication reconciliation study, which included ICU patients and scored MTE at discharge of the ICU. The potential harm of every MTE was estimated with a validated score, where after MTE with potential for harm were indicated as PH-MTE. Predictors for PH-MTE at ICU discharge were identified using LASSO regression. The c statisticprovided a measure of the overall discriminative ability of the prediction model and the prediction model was internally validated by bootstrap resampling. Based on sensitivity and specificity, the cut-off point of the prediction model was determined.

**Results:**

The cohort contained 258 patients and six variables were identified as predictors for PH-MTE: length of ICU admission, number of home medications and patient taking one of the following medication groups at home: vitamin/mineral supplements, cardiovascular medication, psycholeptic/analeptic medication and medication for obstructive airway disease. The c of the final prediction model was 0.73 (95%CI 0.67–0.79) and decreased to 0.62 according to bootstrap resampling. At a cut-off score of two the prediction model yielded a sensitivity of 70% and a specificity of 61%.

**Conclusions:**

A multivariable prediction model was developed to identify patients at risk for PH-MTE after ICU discharge. The model contains predictors that are available on the day of ICU discharge. Once external validation and evaluation of this model in daily practice has been performed, its incorporation into clinical practice could potentially allow institutions to identify patients at risk for PH-MTE after ICU discharge, on the day of ICU discharge, thus allowing for efficient, patient-specific allocation of clinical pharmacy services.

**Trial registration:**

Dutch trial register: NTR4159, 5 September 2013, retrospectively registered.

## Introduction

Discharging patients from an Intensive care unit (ICU) is a high-risk process prone to medication transfer errors (MTE) with a high potential for adverse drug events (ADE) [1]. Possible causes of MTE are multifactorial, relating to the system, the patient and the healthcare staff [2–4]. For instance, the vulnerability of the ICU discharge process may be caused by a lack of standardized discharge procedures, a significant reduction in monitoring of the patient, the number, complexity and acuity of the patient’s medical conditions and finally the involvement of several healthcare providers (multi-professional and inter-specialty) [5,6]. A large Canadian population-based cohort study [7] showed that being a post-ICU-patient was associated with MTE due to unintended discontinuation of chronic home medication at hospital discharge. Moreover, patients who experienced unintended discontinuation of antiplatelet drugs, anticoagulants or statins had a higher risk of adverse events (i.e. death, emergency department visit or emergency hospitalization).

Medication reconciliation by an ICU pharmacist at ICU admission and discharge, reduces MTE and therefore patient harm [1]. Ideally, each hospital pharmacy would have the resources to provide this new medication safety practice to every ICU patient [1,8]. However, especially medication reconciliation at ICU discharge was found not easy to deliver, as it is complex, time-consuming and the time window in the discharge process at the ICU is usually small [1]. Likewise, hospitals are faced with numerous challenges, such as reduced funding, an increasing number of patients, especially those with multi-morbidities and polypharmacy, as well as the demand for a 7-day clinical service [9–11]. These challenges result in a higher amount of work with fewer personnel, urging the need for more innovative approaches to service delivery, for example, prioritization of patients at increased risk of preventable harm [9, 12–15]. Unfortunately, commonly chosen criteria, such as age and number of medications, often fail to properly identify patients at increased risk for harm caused by medication errors, indicating the need for a prediction tool [15].

To date there are no prognostic prediction models available to identify patients at increased risk for potential harmful MTE (PH-MTE) after ICU discharge. However, studies on potential predictors on the admission and discharge at the hospital are available [2,3,16], as well as a few studies on post-ICU patients discharged from the hospital [7,12,17]. For instance, predictors for post ICU patients for unintended drug discontinuation after hospital discharge were a non-academic ICU and patients’ admission without medical diagnosis [7]. Older age, emergency hospitalizations and multiple comorbidities were predictors for unintentional continuation of ICU medication after hospital discharge [17].

The aim of this study was to develop a prognostic multivariable model in patients discharged from the ICU to predict who is at increased risk for PH-MTE after ICU discharge, using predictors of PH-MTE that are readily available at the time of ICU discharge. We used the TRIPOD statement (Transparent Reporting of a multivariable prediction model for Individual Prognosis and Diagnosis) for the reporting of this prediction model [18].

## Materials and methods

### Study design

Data for this study were derived from the Transfer ICU medication reconciliation (TIM) study. This was a prospective before- after-, double center study on the effect of pharmacist led medication reconciliation. The study was performed in the ICU of one general teaching hospital (GTH; Haga, a hospital that provides medical education) and one university hospital (UH; ErasmusMC, a hospital that provides medical education and research facilities, affiliated with Erasmus university), during 8 months in 2013 and 2014 in The Netherlands [19]. Since this study did not affect patients’ integrity, a waiver from the Zuid Holland Medical Ethics committee (METC) (Ref. nr: 12–097) and the Erasmus MC METC (MEC-2014-085) was obtained. This waiver equals ethical approval.

During the pre-intervention period (GTH: January-April 2013 and UH: February-May 2014), conventional care was delivered which was changed to pharmacist led medication reconciliation during the post- intervention period (GTH: May-September 2013, UH: July-October 2014). Patients were included when they used at least one chronic medicine at home and when the ICU stay exceeded 24 hours. Patients were excluded when they died during the ICU stay. We made a “best possible medication history” (BPMH) of medication used at home. Moreover, a best possible general ward medication list 24 hours after ICU discharge (BPML-GW24) was made, based on the BPMH, information in the electronic patient records of the hospital and the ICU, medication prescribed and, whenever necessary, on interviewing the physician on the ward afterwards. Only in the intervention phase were the medication lists shared with patients’ health care providers.

The primary outcomes of the TIM study were the proportions of patients with ≥1 MTE after ICU admission and after ICU discharge.

**Fig 1:**
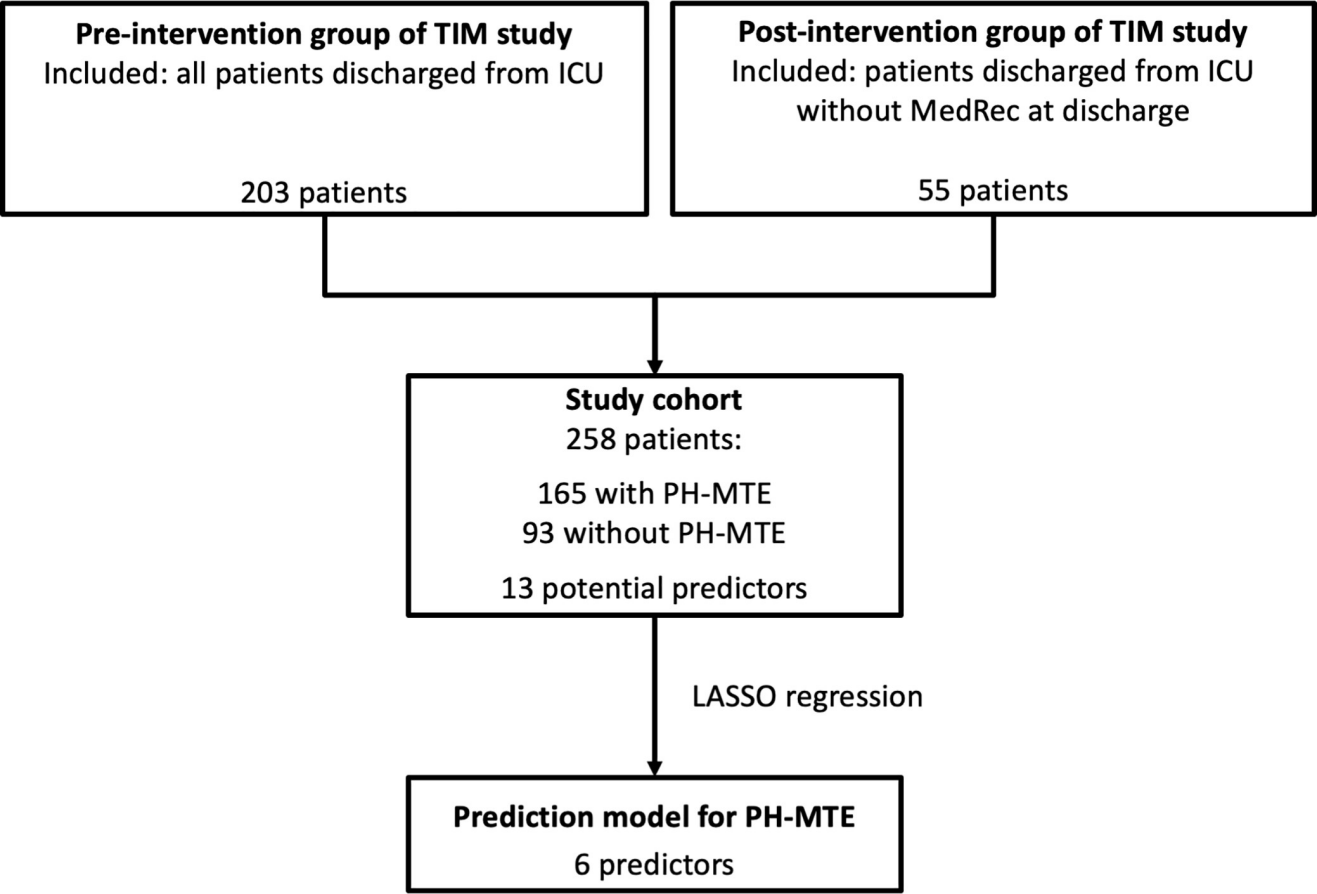
Study design. MedRec = Medication Reconciliation, PH-MTE = potential harmful medication transfer error.

We did not calculate a formal sample size, we used the data of an already available dataset of the TIM study. All eligible patients of the TIM study were included, i.e. all ICU survivors of the pre-intervention phase (203 patients) and the ICU survivors of the intervention period who were missed in the medication reconciliation at ICU discharge (55 patients), as we were interested in predicting who was at risk for PH-MTE without medication verification by a pharmacist at ICU discharge (Fig 1).

The outcome was ≥1 PH-MTE at 24 hours after ICU discharge. An MTE was defined as an unintentional discrepancy between actual prescribing 24 hours after ICU discharge and the BPML-GW24. The likelihood of potential harm of the MTE was estimated, based on a validated pADE score by Nesbit et al. [20]. This score ranged from 0 to 0.6, where pADE = 0 indicated that no harm was expected, while pADE = 0.6 indicated high likelihood of an ADE. For the pADE assessment, the MTEs were presented randomized and blinded to the study period to two assessors: one hospital pharmacist/clinical pharmacologist, and one internist /clinical pharmacologist in training, both with ICU experience. They gave independently from each other a pADE score for each MTE, based on clinical data of the patient. The assessors reached consensus in a meeting in case MTEs received different pADE likelihood scores. We measured a total pADE score for every patient by adding up the individual pADE scores. The patient outcome was divided in the following subgroups: very low likelihood of an ADE (0.01≤ pADE< 0.1), low likelihood of an ADE (0.1≤ pADE< 0.4), medium likelihood of an ADE (0.4≤ pADE< 0.6) and, finally, high likelihood of an ADE (pADE≥0.6). We defined a PH-MTE as an MTE with a pADE≥0.01, meaning that patients with MTE with no likelihood of harm were not selected as an outcome.

### Potential predictors

Potential demographic and clinical predictors for patients suffering PH-MTE at ICU discharge were identified from previous studies on hospital admission and discharge [2,3,7, 12,13,16,21]. As we only wanted to include variables that were easily available on the day of ICU discharge, variables like length of hospital stay, APACHE IV score (Acute Physiology and Chronic Health Evaluation [22]), medication in use after ICU discharge or variables that were influenced by the ICU admission, like renal function, were not included. The selected demographic and clinical potential predictors were: age (continuous as well as log transformed), sex, emergency admission, academic hospital, type of admission (surgical versus medical and emergency versus planned) and duration of ICU stay (continuous as well as log transformed).

Selection of potential medication related predictors was based on different types of studies: studies on hospital admission and discharge [2,3,7,21] and a review on ICU admissions related to ADE [23]. We selected the number of medications on the BPMH as potential predictor (continuous as well as log transformed), as well as the use of the following home medication groups; (1) medication used in diabetes (Anatomic Therapeutic Chemical [ATC] code A10), (2) vitamins and mineral supplements (ATC codes A11 and A12), (3) antiplatelet and anticoagulant agents (ATC code B01), (4) cardiovascular medication (ATC code C), (5) psycholeptic/analeptic (ATC codes N05 and N06), and finally (6) medication for obstructive airway diseases (ATC code R03)[24]. All medication groups were selected based on literature, except for the vitamin/ mineral supplements. We added this group as potential predictor, since this group was representing a broad group of vulnerable patients, for example patients on dialysis (alphacalcidol), patients with addictions or malnutrition (thiamine), patients with cystic fibrosis (vitamin ADEK) or elderly patients (calcium with colecalciferol).

### Statistical analysis

Data analysis was carried out using SPSS statistics, version23 (IBM Corp., Armonk, New York, USA) and R version 3.5.3 (R Core Team (2014). R: A language and environment for statistical computing. R Foundation for Statistical Computing, Vienna, Austria. URL http://www.R-project.org/).

All variables were present, resulting in no missing data. Variables were described as mean (standard deviation [SD]) in case of a normal distribution and median (interquartile range [IQR]) in case of a non-normal distribution. To select the most potent predictors, we used the Least Absolute Shrinkage and Selection Operator (LASSO) algorithm and odds ratios (ORs) were estimated by means of this method [25].

Model building included tenfold cross-validation for choosing the LASSO penalty. In addition, we performed a bootstrap resampling (200 repetitions) of the original set for internal validation. We developed the LASSO models, including the search for the optimal penalty, on bootstrap samples and tested the performance on the original sample to quantify statistical optimism in performance.

To obtain a simplified prediction model, the regression coefficients of the predictive variables were divided by 0.75 and rounded to the next integer for dichotomous variables and to the next half for continuous variables; subsequently, prediction scores according to the prediction model were calculated for every patient. The calculated prediction scores were compared with the observed percentage of patients who experienced PH-MTE. The overall discriminative ability of the prediction model was quantified by a c statistic. The calibration was assessed in a calibration plot. The specificity and sensitivity were determined for several cut-off values of the prediction scores.

## Results

### Patient and medication characteristics

**Fig 2:**
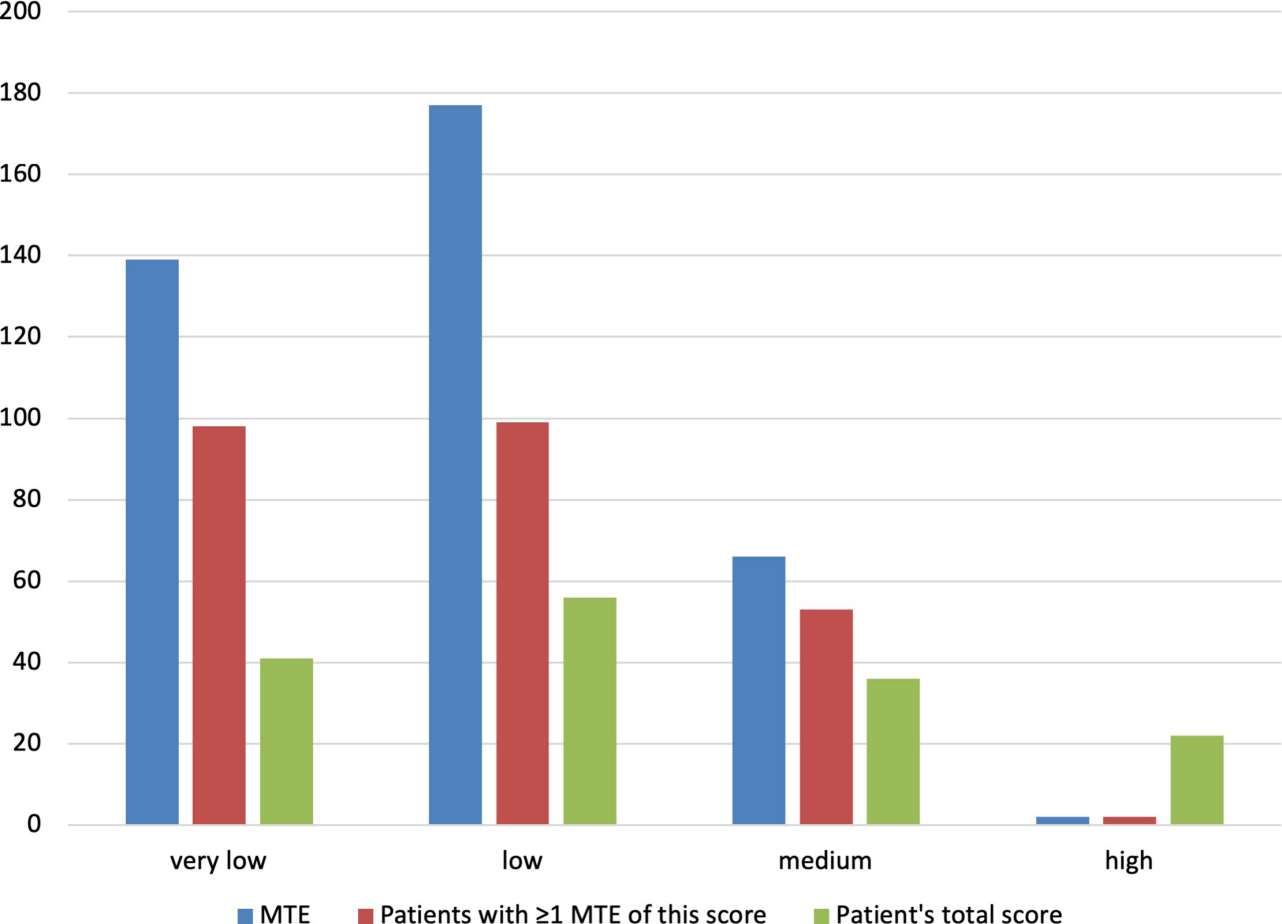
Distribution of pADE scores. The blue bar is the pADE score per MTE, the orange bar the distribution of pADE scores per patient (patients who had at least one MTE with this pADE score) and the gray bar is the distribution of patient’s total pADE score.

The study cohort consisted of 258 patients with a mean age of 60 years (SD 15), with 61% emergency admissions and 41% surgical patients (Table 1). The median number of days in the ICU was 3 (IQR of 2 to 6). The mean number of medications on the BPMH was 5 (IQR of 3 to 9). Twenty one percent of the patients used medication for diabetes at home, 27% minerals/vitamins, mostly for osteoporosis prevention or dialysis vitamins, 46% anticoagulant agents, 67% cardiovascular medication, 26% psycholeptic/analeptic medication and finally 19% used medication for obstructive airway diseases at home. One hundred and sixty-five (64%) patients suffered 383 PH-MTE. Most frequently found MTE after ICU discharge were omissions in home medications (72%). Of these PH-MTE, 66 (17.2%) had a pADE = 0.4 (medium likelihood score) and 2 (0.5%) had a pADE = 0.6 (high ADE likelihood score), resulting in 21.9% of the patients having a medium likelihood of an ADE (0.4≤ pADE<0.6) and 12.7% having a high likelihood of an ADE (pADE≥0.6). Fig 2 shows the distribution of the pADE scores for both the MTEs and the patients. An example of an MTE with pADE = 0.6 was labetalol dose accidentally reduced post ICU from t.i.d. 200mg to o.d. 50mg in a patient who previously suffered a subarachnoidal bleeding, the blood pressure went up to >180mm Hg. An example of pADE = 0.4 was gliclazide t.i.d. 80mg (and metformin t.i.d. 850mg) used at home, not restarted after ICU discharge in a patient with diabetes. An example of pADE = 0.1 was the unintentional continuation of esomeprazole after ICU discharge. An example of pADE = 0.01 was the omission to continue or restart atorvastatin previously used at home.

**Table 1:** Demographic, clinical and drug treatment related characteristics of the patient population, potential predictors.

Patient characteristics	With PH-MTE (n = 165)	Without PH-MTE (n = 93)	OR (95% CI)	P
Demographic and clinical characteristics				
Age, mean (SD), y	60 (14)	59 (17)	1.00 (0.99–1.02)	0.74
Female sex, n (%)	56 (34%)	29 (31%)	1.13 (0.66–1.95)	0.65
Emergency admission	106 (64%)	51 (55%)	1.48 (0.88–2.48)	0.14
Academic, n (%)	103 (62%)	60 (65%)	0.91 (0.54–1.55)	0.74
Surgical, n (%)	62 (38%)	45 (48%)	0.93 (0.47–1.83)	0.09
Days in ICU, median (IQR)	3 (2–7)	3 (2–4)	1.03 (1.00–1.07)	0.05
Drug treatment related characteristics				
No medications on BPMH, median (IQR)	6 (4–10)	4 (2–7)	1.16 (1.08–1.25)	<0.001
Patients with potential high-risk home medication (ATC)				
Medication used in diabetes (A10)	36 (22%)	18 (19%)	1.16 (0.62–2.19)	0.64
Vitamins/mineral supplements (A11, A12)	55 (33%)	15 (16%)	2.60 (1.37–4.93)	0.003
Antiplatelet/anticoagulant agents (B01)	79 (48%)	39 (42%)	1.27 (0.76–2.12)	0.36
Medication cardiovascular tract (C)	121 (73%)	52 (56%)	2.17 (1.27–3.70)	0.004
Psycholeptic/analeptic medication (N05, N06)	52 (32%)	14 (15%)	2.60 (1.35–5.01)	0.004
Medication for obstructive airway diseases (R03)	39 (24%)	9 (10%)	2.89 (1.33–6.27)	0.006

IQR = interquartile range, OR = odds ratio, SD = standard deviation, BPMH = Best possible medication history, ATC = Anatomical Therapeutic Chemical code [24] A11 = vitamins, A12 = mineral supplements, C = cardiovascular medication (digoxin, antihypertensives, diuretics, vasoprotectives, beta-blocking agents, calcium channel blocking agents, agents acting on the RAS or lipid modifying agents), N05 = psycholeptic medication N06 = analeptic medication, R03 = Medication for obstructive airway diseases.

### Developing the prediction model

Six out of 13 predictors were identified by LASSO regression model: length of ICU admission, number of medications of the BPHM and patients taking one of the following medication groups at home: vitamin/mineral supplements, cardiovascular medication, psycholeptic/analeptic medication and medication for obstructive airway disease. The odds of PH-MTE were two to three-fold higher for the final predictors, as can be seen in Table 1. After applying LASSO regression, OR were 1.01- to 1.11-for PH-MTE (Table 2). After calculating the prediction score, the median score based on the adjudicated points was 2.5 (IQR of 1.5 to 3.5) for patients with PH-MTE compared to 1.5 (IQR of 1 to 2) for patients without PH-MTE. Four out of 11 patients (36%) patients with a prediction score = 0 experienced a PH-MTE compared to 11 out of 12(92%) with a prediction score ≥5 (Table 3). The model predicted best at a cut-off point of 2 with a sensitivity of 70% and a specificity of 61%. With this cut-off value, 60% of the ICU patients were selected as being at high risk for PH-MTE, indicating a 40% reduction in workload and an estimated 1–2 hours (2–3 patients) per day to spend on medication reconciliation at ICU discharge for our GTH and 2–3 hours (3–5 patients) for our UH, in the case this tool were to be implemented.

**Table 2:** Selected predictors on PH-MTE, regression coefficients, adjusted ORs and points for prediction rule.

Variables prediction model		B	OR	Points^#^
**log(Days in ICU)**		0.046	1.05	
	**1–2 days**			0
	**3–6 days**			0.5
	**7–20 days**			1.0
	**21 or more days**			1.5
**Number of medications on BPMH**		0.0097	1.01	
	**0–4 medications**			0
	**5–9 medications**			0.5
	**10–14 medications**			1.0
	**15–19 medications**			1.5
	**20 or more medications**			2.0
Vitamins/mineral supplements used at home (A11, A12*)		0.067	1.07	1.0
Cardiovascular home medication (C*)		0.080	1.08	1.0
Psycholeptic /analeptic home medication (N05, N06*)		0.107	1.11	1.0
Medication for obstructive airway diseases (R03*)		0.101	1.11	1.0

B = Regression coefficients, OR = Odds ratio, BPMH = Best possible medication history

^#^ = Points for the simplified prediction rule derived from the regression coefficient.

* = Anatomical Therapeutic Chemical codes [24]: A11 = vitamins, A12 = mineral supplements, C = cardiovascular medication (digoxin, antihypertensives, diuretics, vasoprotectives, beta-blocking agents, calcium channel blocking agents, renin-angiotensin system blocking agents or lipid modifying agents), N05 = psycholeptic medication N06 = analeptic medication, R03 = Medication for obstructive airway diseases.

**Table 3:** Prediction scores and cut-off values for high risk of potential harmful medication transfer errors (PH-MTE) or not (No-PH-MTE).

Prediction score*	**PH-MTE**	**No PH-MTE**	
**0**	4 (36%)	7 (64%)	
**0.5**	9 (50%)	9 (50%)	
**1**	15 (48%)	16 (52%)	
**1.5**	21 (46%)	25 (54%)	
**2**	24 (62%)	15 (38%)	
**2.5**	20 (67%)	10 (33%)	
**3**	24 (80%)	6 (20%)	
**3.5**	12 (86%)	2 (14%)	
**4**	9 (90%)	1 (10%)	
**4.5**	11 (92%)	1 (8%)	
**5**	5 (100%)	0 (0%)	
**5.5**	5 (100%)	0 (0%)	
**6**	6 (86%)	1 (14%)	
**Cut-off values**	**PH-MTE**	**No PH-MTE**	
**1**			
**Score < 1**	13	**16**	specificity = 17%
**Score ≥1**	**152**	77	sensitivity = 92%
**2**			
**Score < 1.5**	28	**32**	specificity = 34%
**Score ≥ 1.5**	**137**	61	sensitivity = 83%
**2**			
**Score<2**	49	**57**	specificity = 61%
**Score ≥ 2**	**116**	36	sensitivity = 70%
**2.5**			
**Score < 2.5**	73	**72**	specificity = 77%
**Score ≥2.5**	**92**	21	sensitivity = 56%
**3**			
**Score < 3**	93	**82**	specificity = 88%
**Score ≥ 3**	**72**	11	sensitivity = 43%

*values are the number (%) of patients with a given score.

The c statistic of the final prediction model was 0.73 (95%CI 0.67–0.79) and decreased to 0.62 after bootstrap validation. S1 File shows the calibration plot.

**Fig 3:**
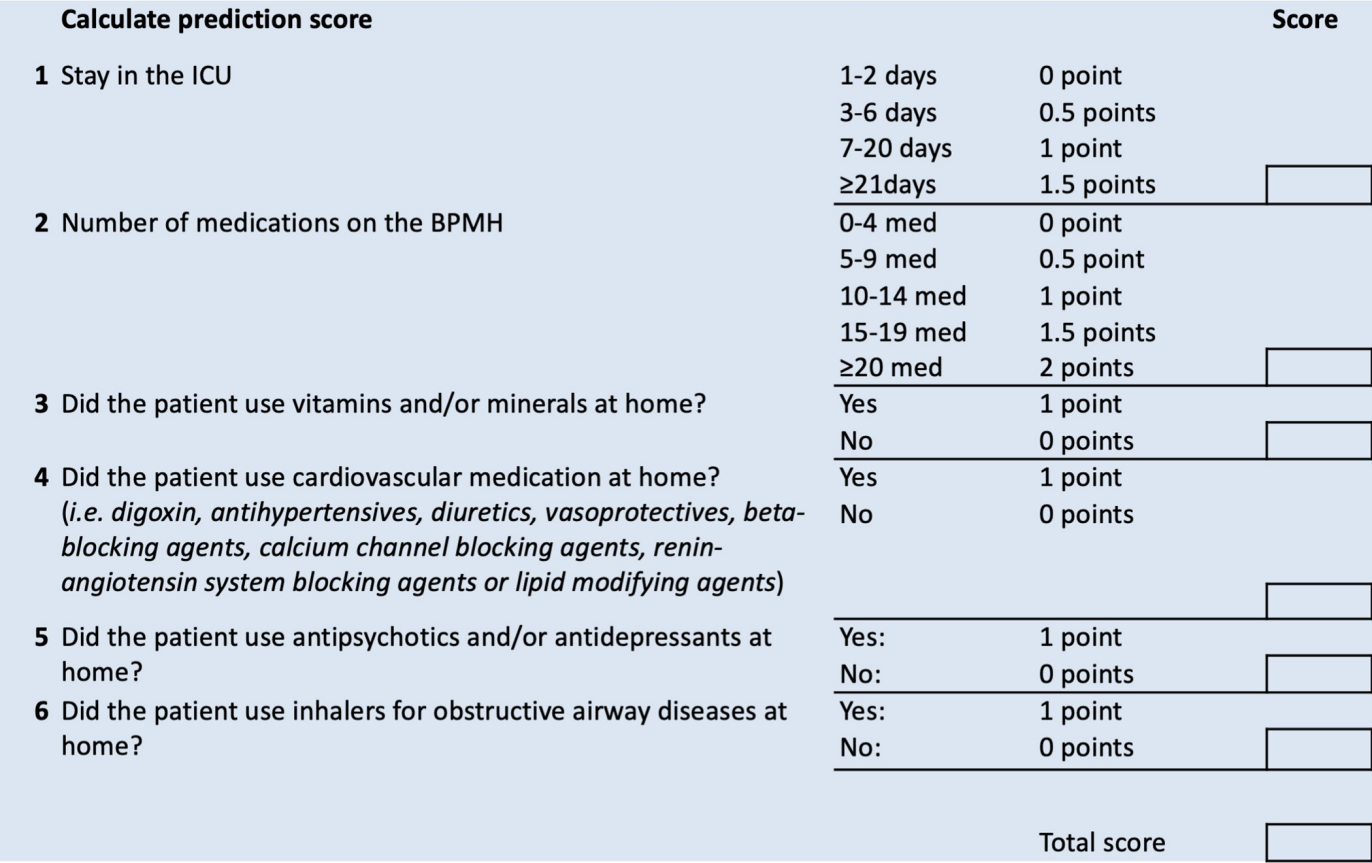
Patient’s prediction score calculator for potential harmful medication transfer errors.

Fig 3 shows the form that can be used in daily practice by the medical staff prior to the ICU discharge process to calculate the patient’s prediction score for PH-MTE.

## Discussion

This is the first study that developed a prediction model for PH-MTE at ICU discharge. The developed model contained six predictors that can easily be obtained at the moment the ICU discharge process starts: length of ICU admission, number of medications in use at home and patients taking one of the following medication groups at home: vitamin and mineral supplements, cardiovascular medication, psycholeptic/analeptic medication and medication for obstructive airway disease. The c-statistic of the model was 0.73 (95%CI 0.67–0.79) and decreased to 0.62 after bootstrapping. In addition, sensitivity and specificity of the prediction model was best at a cut-off value of two, namely 70% and 61%. With this cut-off value 60% of the ICU patients were selected as being at increased risk for PH-MTE. This 40% reduction in workload is substantial, increasing the probability that implementation of medication reconciliation at ICU discharge will become feasible.

Our study is in line with other studies seeking to develop prediction models in order to prioritize pharmaceutical care [14,26].

The finding that emergency admissions and age were not associated with MTE is in line with results from Bell et al. [12]. In contrast with their results, we did not find that academic hospitals and patients with medical diagnoses were associated with more MTE. In addition, our study indicated that the number of medications on the BPMH and the length of stay in the ICU were associated with more MTE, whereas Bell et al did not. These differences may be due to differences in study population, setting and outcome. In line with our findings, studies on predictors for MTE on hospital transfers found the number of medications on the BPMH [2,7,21] and the length of hospital stay [7] as a predictor for MTE. The medication groups that predict PH-MTE were in line with the literature on hospital transitions and the ICU ADE review [23]. Exceptions were the use of antidiabetics and the use of antiplatelet/ anticoagulant agents. A possible explanation for this difference is that we used home medication as predictors and not medication started in the hospital, which for anti-diabetics and anticoagulants probably would have predicted better.

The most important strength of our study was the simplicity of the developed prediction model. The predictors used in our prediction rule can easily be obtained in daily practice. Another strength of our study was the detailed medication information we obtained which made it possible to assess home medication groups as potential predictors for PH-MTE. Moreover, we used the TRIPOD reporting checklist of prediction models, making it possible to reveal strengths and weaknesses, thereby facilitating the interpretation of our study [18]. In addition, we only looked into predicting PH-MTEs instead of MTEs, which are clinically relevant MTEs, needing additional attention.

Some limitations need to be addressed as well.

First, we were not able to include a few potential predictors found in literature, e.g. a comorbidity index score, like Charlson comorbidity index [13] or individual comorbidities, like renal insufficiency. The main reason we did not include these predictors was that these data were not available in the TIM database. Besides, as we wanted to design a prediction tool that was user friendly, these predictors were less suitable since they are not always documented properly [14] and part of these are subject to change around ICU admissions, making them difficult to define and apply as intended. Instead of co-morbidities, we selected several types of home medications as potential predictors, since home medication information is easily available and can be seen as an indication of chronic disease. I.e. insulin or metformin used at home indicates that the patient has diabetes.

Second, the number of available potential predictors in our dataset was high and our sample size was relatively small, which could have resulted in overfitting. In order to prevent this, we made a strict selection of potential predictors based on literature. We hence used penalization methods to limit the risk of over-fitting [26].

Third, we did not have an external cohort to validate our prediction model.

Fourth, our study outcome was PH-MTE 24 hours after ICU discharge, instead of actual preventable ADE and the extent of long-term harmful effects of the MTE was not determined, since we didn’t follow up after hospital discharge. Likewise, measuring ADEs occurring due to the MTE was not feasible, since we frequently alerted the physician on the MTEs made, as this was part of the MTE assessment. Alerting the physician on the MTEs made was also found necessary for ethical reasons.

Likewise, we chose a low pADE of 0.01 (very low likelihood of an ADE) as a cut-off because of the nature of MTE at ICU discharge: an MTE at ICU discharge at the ward has the potential to continue, therefore even MTE with low likelihood for harm (pADE = 0.01) can, due to their potential of prolonged exposure, lead to harm in the end. This was clearly demonstrated by Bell et al, who found omissions of “home statins” (pADE score 0.01) due to ICU stay, lead to a higher risk of adverse events after hospital discharge [7].

Finally, the c-statistic decreased by means of bootstrapping, which indicates that the results obtained from our model may not be stable, meaning that other studies are necessary to further develop and validate the model. In addition, the specificity, i.e. the ability to correctly identify patients without PH-MTE (true negative rate) was not as good as one would hope. However, the ICU discharge is a high-risk process making sensitivity more important than specificity [5,6]. Noteworthy, we found that 36% of the patients with a prediction score of zero, had PH-MTE nevertheless, clearly indicating that this tool by itself will miss patients with PH-MTE. This makes sense since our prediction model contains predictors related to the patient and not related to the system or healthcare staff, which are other known causes for MTE [2–4]. So, for successful elimination of MTE after ICU discharge, this tool needs to be combined with other methods to address the other causes for MTE, for example avoiding ICU discharge in the “after hours” [27], using transition programs [28], and/or using a standardized handover process for ICU discharge using both oral and written formats for reporting [27].

Prospective validation of our risk prediction model should be the next step in future research. Ideally in an independent set of patients from other hospitals. The predictive performance of the risk prediction model should be further optimized and tested in daily practice, as part of the patient transfer at ICU discharge. Moreover, ideally, the risk prediction model should be incorporated into the ICU electronic information system, generating automatic risk-evaluation upon ICU discharge. Finally, to further optimize the pharmaceutical care of the vulnerable post ICU patient, more insight into which medications lead to the most harmful and/or frequent MTE is needed.

## Conclusions

The discharge from the ICU is a high-risk process, leading to numerous PH-MTE after ICU discharge. We developed a model to predict patients at risk for PH-MTE at ICU discharge. Once external validation and evaluation of this model in daily practice has been performed, its incorporation into clinical practice could potentially allow institutions to identify patients at risk for PH-MTE after ICU discharge on the day of ICU discharge, thus allowing for efficient, patient-specific allocation of clinical pharmacy services.

## Supporting information

S1 File Calibration plot. (TIFF)

S2 File Dataset patients at ICU discharge with and without PH-MTE. (XLSX)

## Abbreviations

APACHE IV: acute physiology and chronic health evaluation IV
ATC: Anatomical Therapeutic Chemical (a medication classification system)
BPMH: best possible medication history
BPML-GW24: best possible general ward medication list 24 hours after ICU discharge
ICU: intensive care unit
LASSO: Least Absolute Shrinkage and Selection Operator
MTE: medication transfer error
OR: Odds Ratio
pADE: potential adverse drug event
PH-MTE: MTE after ICU discharge with the potential to cause harm
TIM: Transfer ICU and Medication reconciliation program
