# Supplementary figures and images for: Development of a multivariable prediction model for identification of patients at risk for medication transfer errors at ICU discharge
Source: PLoS One. 2019 Apr 30;14(4):e0215459. doi: 10.1371/journal.pone.0215459 (PMC6490883; doi:10.1371/journal.pone.0215459)

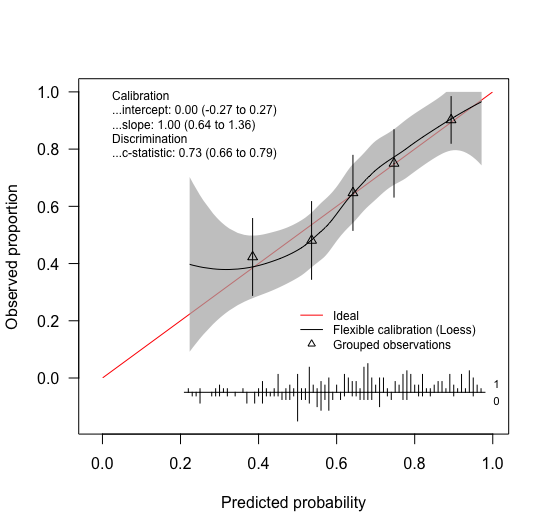

Supplement: S1 File — (TIFF) [file pone.0215459.s001.tiff]
